# Survival after Consecutive Myocardial Infarction and Stroke: A Case Report

**DOI:** 10.15388/Amed.2024.31.1.14

**Published:** 2024-02-27

**Authors:** Kristina Ratautė, Greta Burneikaitė, Jolita Badarienė

**Affiliations:** 1 Faculty of Medicine, Vilnius University, Vilnius, Lithuania; 2 Clinic of Cardiac and Vascular Diseases, Institute of Clinical Medicine, Faculty of Medicine, Vilnius, Lithuania

**Keywords:** myocardial infarction, stroke, cerebral infarction, mortality, miokardo infarktas, insultas, smegenų infarktas, mirtingumas

## Abstract

**Background:**

Myocardial infarction and stroke are prevalent and potentially fatal urgent medical conditions. Stroke as a subsequent cardiovascular event after the myocardial infarction significantly decreases the odds of survival for the patient.

**Clinical case:**

We report a case of a 48-year-old man admitted to Vilnius University Hospital Santaros Klinikos due to an ST-segment elevation myocardial infarction. Patient also experienced a cardioembolic cerebral infarction on the tenth day in the hospital. The patient survived this dual infarction, his general condition improved and he was discharged to the rehabilitation center.

**Discussion and Conclusions:**

Cardiovascular diseases are the most common cause of death in the world. Stroke, as a complication of myocardial infarction, affects 0.76–3.2% of patients and demonstrates an increasing incidence trend. In such a dual infarction, in-hospital mortality can be as high as 18–41%. It is hopeful that targeted research and evidence-based prevention with treatment can improve outcomes of concomitant myocardial infarction and stroke.

## Abstract (it)

**Apžvalga:**

Miokardo infarktas ir insultas yra plačiai paplitę, skubios medicininės pagalbos reikalaujantys, ir potencialiai mirtini sveikatos sutrikimai. Insultas po miokardo infarkto, kaip vėlesnis širdies ir kraujagyslių sistemos įvykis, ženkliai sumažina paciento išgyvenamumą.

**Klinikinis atvejis:**

Pristatome 48 metų vyro, hospitalizuoto į Vilniaus universiteto ligoninės Santaros klinikas dėl miokardo infarkto su ST segmento pakilimu, klinikinį atvejį. Pacientas taip pat patyrė kardioembolinį smegenų infarktą dešimtą hospitalizacijos dieną. Pacientas išgyveno dvigubą infarktą, jo bendra būklė pagerėjo, ir jis buvo išrašytas į reabilitacijos skyrių.

**Diskusija ir išvados:**

Širdies ir kraujagyslių ligos yra dažniausia mirties priežastis pasaulyje. Insultą, kaip miokardo infarkto komplikaciją, patiria 0,76-3,2% pacientų, su stebimu vis didėjančiu dažniu. Tokio dvigubo infarkto atveju mirtingumas ligoninėje gali siekti net 18–41%. Tiksliniai tyrimai ir įrodymais pagrįsta prevencija bei gydymas galėtų pagerinti miokardo infarkto ir jo pasekoje pasireiškiančio insulto klinikines išeitis.

## Introduction

Myocardial infarction (MI) and stroke are prevalent and potentially fatal urgent medical conditions. More than 7 million individuals experience MI and about 16 million develop a stroke each year worldwide [1,2]. MI and stroke share major risk factors, such as obesity, dyslipidaemia, hypertension, diabetes, and smoking [3]. According to different articles, the occurrence of stroke as a complication after MI is noted for 0.76–3.2% patients, with an increasing trend [4-7]. More than 50% of strokes occur on the first day after admission for MI, and nearly 90% within the first 5 days [8]. Based on different articles, 30–89% of strokes after MI are ischemic [4,5,8,9]. Two thirds of in-hospital ischemic strokes following MI are cardioembolic origin [8,9]. The most common disorders associated with a high risk of cardioembolism include recent MI, atrial fibrillation, and other cardiac conditions [5-10]. Stroke as a complication of MI is devastating, as it significantly increases morbidity and mortality of the patient. In this report, we present myocardial infarction with stroke and discuss the potential survival odds after such dual infarction.

## Clinical case

**Fig. 1:**
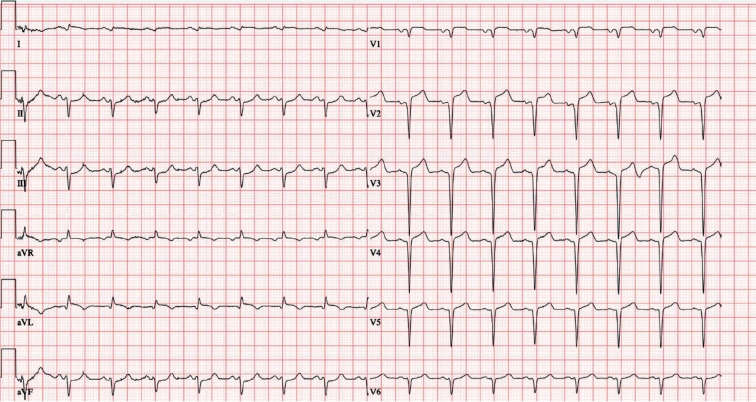
The electrocardiogram showing an ST-segment elevation myocardial infarction

A 48-year-old man presented with sudden intense epigastric pain and shortness of breath. The patient called an ambulance 16 hours after the onset of pain. An electrocardiogram showed an ST-segment elevation myocardial infarction (STEMI) (Fig. 1) and the level of troponin I was significantly elevated (99185 ng/L). Emergency coronary angiography was performed, revealing acute occlusion of left anterior descending (LAD) artery, along with critical stenosis of the right coronary artery (RCA) and left circumflex (LCx) artery (Table 1). Percutaneous coronary intervention (PCI) was performed on the LAD with stent placement.

**Table 1:** Coronary angiography showed occlusion of left anterior descending artery, critical stenosis of the right coronary artery, and critical stenosis of the left circumflex artery

Right coronary artery	%	Left anterior descending artery	%	Left circumflex artery	%
Segment 1	-	Segment 6	100	Segment 11	90
Segment 2	90	Segment 7	100	Segment 12	-
Segment 3	80	Segment 8	-	Segment 13	-
Segment 4	-	Segment 9	-	Segment 14	-
Segment 5	-	Segment 10	-	Segment 15	-

The patient’s medical history was collected. The patient claimed to have no concurrent illnesses and not to be taking any medication. He smoked half a pack a day for many years, currently does not smoke, and consumes alcohol once every 1–2 months. The patient’s cousin from the maternal side experienced a myocardial infarction at the age of 42 while sleeping.

Patient’s physical examination after the intervention: blood pressure 110/70 mmHg, heart rate 104 beats/min, heart sounds rhythmic without murmurs, respiratory rate 16 breaths/min, vesicular breathing sounds with audible moist rales, oxygen saturation 98% with 5 L/min oxygen via nasal cannula, abdomen soft and painless, and no signs of oedema. Laboratory tests showed normal complete blood count, elevated glucose (13.53 mmol/L) and increased glycated hemoglobin (8.9%), dyslipidaemia (triglycerides 2.68 mmol/L, total cholesterol 6.71 mmol/L, low-density lipoprotein cholesterol 4.09 mmol/L, non-high-density lipoprotein cholesterol 5.32 mmol/L, lipoprotein (a) 369 nmol/L), elevated B-type natriuretic peptide (445.3 ng/L), elevated liver enzymes (aspartate aminotransferase 1363 U/L and alanine aminotransferase 155 U/L), normal creatinine (90 µmol/L) with glomerular filtration rate (87 mL/min/1.73 m2), normal range of electrolytes. The echocardiogram showed left ventricular (LV) hypertrophy, an LV ejection fraction of 25–30%, impaired LV relaxation, and the presence of an apical aneurysm.

**Fig. 2:**
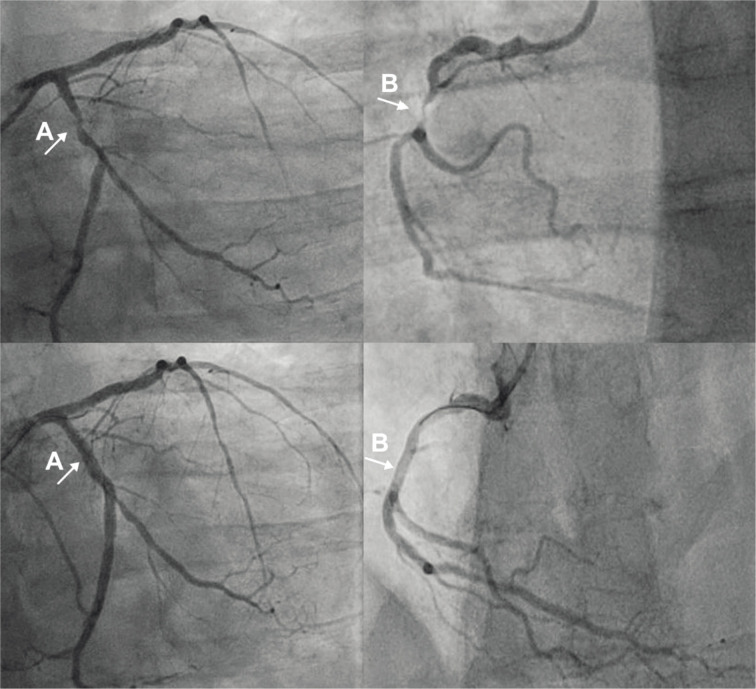
Percutaneous coronary intervention procedures performed on the left circumflex (A) and right coronary (B) and arteries

The patient received aspirin 100 mg, ticagrelor 90 mg twice per day, atorvastatin 40 mg (the dose was reduced according to the patient’s elevated liver enzymes), metoprolol 150–200 mg (depending on the heart rate), ramipril 2.5 mg, spironolactone 25 mg, dapagliflozin 10 mg, short-acting insulin as needed, and omeprazole 20 mg. Additionally, torasemide 50 mg was added to alleviate patient’s nocturnal dyspnea caused by heart failure. Further PCI procedures were performed on the LCx and RCA arteries (Fig. 2). However, despite interventions, nocturnal dyspnea persisted. The second echocardiogram revealed several thrombi at the apex of the LV, one of which had some mobility, as well as fluid up to 13 mm in the pericardial cavity. Due to the thrombus triple antithrombotic therapy was initiated – ticagrelor was discontinued, and clopidogrel 75 mg with rivaroxaban 20 mg were administered alongside aspirin. For the pericardial effusion intravenous furosemide 80 mg was started. After a couple of days dyspnea resolved.

**Fig. 3:**
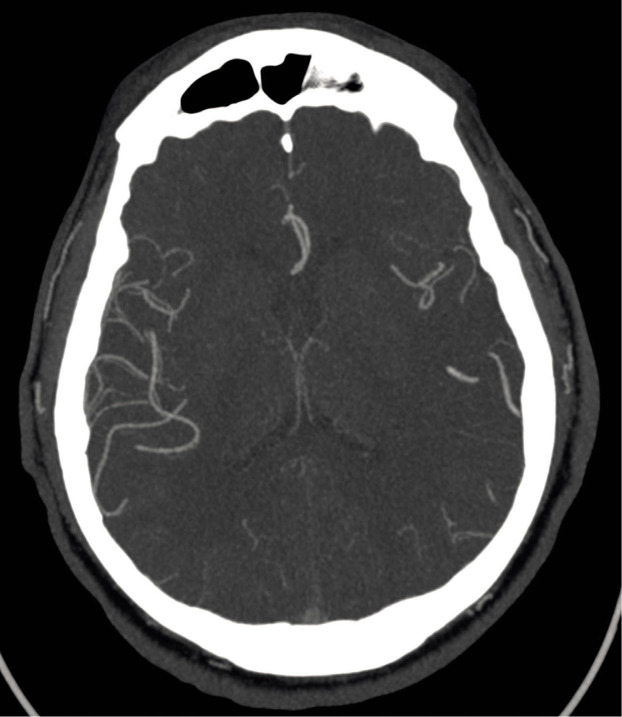
Head computed tomography scan showing a cerebral infarction in the left middle cerebral artery territory with occlusion and impaired distal blood flow

On the morning of the patient’s tenth day in the hospital, the patient felt right-sided limb weakness and fell to the ground, presenting with sensorimotor aphasia and right hemiparesis. A head computed tomography (CT) scan and CT angiography were performed, confirming a cerebral infarction in the left middle cerebral artery (MCA) territory with M2 occlusion and impaired distal blood flow (Fig. 3). Intravenous thrombolysis was contraindicated due to the evening administration of rivaroxaban. Mechanical thrombectomy was performed, achieving complete perfusion. Rivaroxaban administration was suspended for five days. With treatment and rehabilitation patient’s condition gradually improved, with observable progress in speech and right side limb function. However, after six days, the patient noticed fresh blood traces in the stool and due to the high risk of bleeding aspirin was discontinued. The following day, there were no blood traces in the stool, and the fecal occult blood test was negative.

The patient’s general condition improved and he was discharged to the rehabilitation department. The patient will continue treatment with clopidogrel, rivaroxaban (for six months due to LV thrombi), atorvastatin 80 mg (liver enzymes were within normal limits at discharge), ramipril, metoprolol, spironolactone, torsemide 10 mg, omeprazole 20 mg, empagliflozin 10 mg, and metformin 850 mg three times per day. It is advised to refrain from smoking, maintain a balanced diet, and engage in regular physical activity. The target blood pressure is 120–129/80 mmHg, heart rate 55–65 beats/min, low-density lipoprotein cholesterol <1.4 mmol/L, and fasting blood glucose 7–8 mmol/L. Additionally, an outpatient consultation with a cardiologist is recommended due to a family history of early death from a myocardial infarction and an elevated lipoprotein (a) level, as well as a outpatient consultation with a neurologist, and a scheduled colonoscopy to determine the cause of bleeding.

## Discussion

Cardiovascular diseases are the most common cause of death, responsible for more than a third of all deaths globally [1]. The primary cause of death in all countries is ischemic heart disease, which commonly manifests as a myocardial infarction [1,11,12]. In developed countries, the mortality of STEMI patients varies between 4% and 12% in the hospital [11]. However, it is estimated that the mortality is 50% lower than it was prior to the era of advanced cardiovascular care [13]. Advances in therapy and preventive strategies have significantly reduced mortality in the recent decades [1,11, 13].

Stroke is the second most frequent cause of death worldwide [14,15]. In-patient mortality rates from stroke range between 5% and 15% [16]. Cardioembolic cerebral infarction is associated with higher mortality as compared to other stroke types [10,17]. In the recent decades, the mortality of stroke is also decreasing due to healthcare improvements [2,14].

Stroke, as a complication of MI, affects 0.76–3.2% of patients and demonstrates an increasing incidence trend [4-7]. Patients with STEMI are at a higher risk of stroke compared with patients presenting with non-ST-segment elevation myocardial infarction [18]. The majority (30–89%) of strokes after MI are ischemic [4,5,8,9] and 12–20% of strokes after STEMI are hemorrhagic [4,5]. The cause of stroke may not be determined in as many as 60% of cases [4]. More than half of strokes occur on the first day after admission for MI, and nearly 90% within the first 5 days [8]. Hemorrhagic strokes tend to happen within two days of primary PCI, while ischemic strokes occur beyond two days [5]. The leading cause of ischemic strokes in the hospital after MI is cardioembolism, responsible for 60% of in-hospital ischemic strokes [8,9]. Cardiac conditions after MI, especially atrial fibrillation, can lead to intracardiac thrombus formation and be responsible for emboli migrating from the heart [5-10]. The echocardiogram to detect intracardiac thrombi and electrocardiogram for atrial fibrillation diagnosis can be done in order to adequately evaluate and manage patients [7]. Cardioembolic origin ischemic stroke can be preventable with guideline-based anticoagulation [19]. In this case report, the patient had a STEMI and consecutive ischemic cardioembolic stroke, which is the most common type after MI. However, the in this case, stroke occurred after more than first 5 days. Our patient’s echocardiogram revealed intracardiac thrombi, and he received anticoagulation treatment, but stroke occurred nevertheless.

Stroke is a devastating occurrence after MI, as it is closely associated with patient’s decreased survival. A successive stroke in MI patients is associated with significantly higher in-hospital and long-term mortality. Based on different articles, in such a dual infarction, in-hospital mortality can be as high as 18–41%, where up to 4 out of 10 individuals may die after concurrent MI and stroke [4,6,7]. This mortality is over 3 times higher than after STEMI and 1.5 times higher than after stroke. The patient in this case report, however, survived the dual infarction. Long term mortality is estimated to be 33–35% [4,9]. Furthermore, about half of patients with both STEMI and stroke are discharged to a nursing facility, and only 60% of them are eventually discharged home [4]. Additionally, stroke is also associated with longer hospital stay, increased hospital readmissions, and consecutively higher cost, which contribute additional burdens to the patient and the whole healthcare system as well [5-7].

Predictors of subsequent cardiovascular events include older age, hypertension, diabetes, chronic kidney disease, stroke, atrial fibrillation, intracardiac thrombus, heart failure, and deviation from guideline-based treatment [6-8,10,20]. Our patient had a higher risk of stroke following MI due to having hypertension, diabetes, LV thrombi, and heart failure, and still remains at a heightened risk for future cardiovascular events. Recurrences may be preventable by appropriate treatment during the acute phase and strict follow-up care [10].

Improvements in prevention and treatment, including healthier lifestyle, prioritizing percutaneous coronary intervention, modern antithrombotic and anticoagulation therapies, and increased usage of secondary prevention, have contributed to the continuous reductions of MI mortality [1,11]. Effective primary prevention strategies, improvements in hospital care after occurrence of a stroke, and long-term preventive treatment can further reduce mortality rates after stroke [14,16,17]. It is hopeful that targeted research and evidence-based prevention with treatment can improve outcomes of concomitant MI and stroke as well.

## Conclusions

This clinical case demonstrates that one cardiovascular event can sometimes be followed by another, and a myocardial infarction can concurrently occur with a stroke. Dual infarction can be devastating, significantly increasing the already high mortality risk for the patient. There is hope that ongoing medical research with constantly improving prevention and treatment strategies can improve outcomes for patients experiencing both myocardial infarction and stroke during the same hospitalization period.
